# Guanidinoacetic Acid and Its Impact on the Performance, Carcass and Meat Quality of Growing and Finishing Nellore Cattle †

**DOI:** 10.3390/vetsci12050425

**Published:** 2025-04-30

**Authors:** Letícia Carolina Bortolanza Soares, Leticia Kim Huang, Germán Darío Ramírez-Zamudio, Murilo Soler de Magistri, Joao Marcos Bovetto de Campos Valim, Vinicius Laerte Silva Herreira, Patricia Maloso Ramos, Carl Robertson Dahlen, Nara Regina Brandão Cônsolo, Saulo Luz Silva, Paulo Roberto Leme

**Affiliations:** 1 Department of Animal Science, School of Animal Science and Food Engineering, University of Sao Paulo, Pirassununga 13635-900, SP, Brazil; leticiabortolanza@usp.br (L.C.B.S.); leticiahuang@usp.br (L.K.H.); murilomagistri@usp.br (M.S.d.M.); ramospm@usp.br (P.M.R.); sauloluz@usp.br (S.L.S.); prleme@usp.br (P.R.L.); 2 Department of Animal Nutrition and Production, College of Veterinary Medicine and Animal Science, University of Sao Paulo, Pirassununga 13635-900, SP, Brazil; joaobovetto@usp.br (J.M.B.d.C.V.); nara.consolo@usp.br (N.R.B.C.); 3 Department of Animal Bioscience, School of Animal Science and Food Engineering, University of Sao Paulo, Pirassununga 13635-900, SP, Brazil; vinicius.laerte@usp.br; 4 Center for Nutrition and Pregnancy, Department of Animal Sciences, North Dakota State University, Fargo, ND 58108, USA; carl.dahlen@ndsu.edu

**Keywords:** beef cattle, carcass, feedlot, pasture

## Abstract

This study evaluated the effects of guanidinoacetic acid (GAA) supplementation during the growing and finishing phases on Nellore bulls’ performance, carcass traits, and meat quality. Fifty-two Nellore bulls were randomly assigned to four treatments: control (CON, without GAA) and three GAA levels (3, 6, and 9 g/kg dry matter [DM] in the growing supplement; 0.3, 0.6, and 0.9 g/kg DM in the finishing diet). During the 280-day growing phase, bulls were kept in paddocks, while in the 74-day feedlot finishing phase, they were housed in collective pens. Body weight, average daily gain, and carcass ultrasound measurements were recorded in both phases. Feed intake was recorded daily during finishing, and carcass traits were evaluated post-slaughter. GAA supplementation during the growing phase did not affect performance or ultrasound carcass traits *(p* ≥ 0.12). In the finishing phase, increasing GAA doses linearly reduced body weight gain (*p* = 0.03) without affecting carcass traits assessed by ultrasound or post-slaughter (*p* ≥ 0.10). GAA supplementation linearly reduced cooking loss (*p* = 0.02) without influencing other meat quality parameters (*p* ≥ 0.11). It is concluded that, under the conditions of this study, GAA supplementation did not affect performance or carcass traits, except for reduced weight gain at the highest dose, but reduced water loss from cooked meat.

## Simple Summary

The pasture-based growing phase is the longest stage in a bull’s life in tropical beef production systems. It plays a fundamental role in muscle development, influencing growth and meat production later during the finishing phase. This study evaluated the effects of a compound called guanidinoacetic acid (GAA), which assists in energy production and muscle development. Although GAA has shown positive effects in non-ruminant animals, such as pigs and poultry, little is known about its impact on cattle. In this research, 52 young Nellore bulls were divided into four groups and received different amounts of GAA during the pasture-based growing and feedlot finishing phases. The growth, feed intake, fat and muscle development, and meat quality were evaluated. The results showed that GAA did not improve performance or carcass traits during the pasture-based growing phase. In the feedlot, animals receiving higher doses of GAA gained less weight, although carcass characteristics remained similar. One positive finding was that GAA reduced water loss from meat during cooking, which may enhance juiciness. In summary, GAA did not improve performance or carcass traits but may offer a small benefit to meat quality in tropical beef production systems.

## 1. Introduction

In beef cattle production systems, the growing phase, from weaning to the beginning of the finishing period, is characterized by the animals’ great growth potential and lean tissue deposition [1]. However, in tropical and subtropical areas, the growing stage is predominantly carried out in pasture-only systems, where limited feed availability during the dry season and the low nutritional quality of pastures, especially protein content, restrict optimal tissue and muscle growth [2,3].

The energy required for maintaining and growing skeletal muscle is obtained through three main processes: aerobic oxidation (glucose, glycogen and fatty acids), glycolysis, and the phosphocreatine/creatine system [4]. The latter is the fastest ATP synthesis and recycling source, although it has a lower phosphocreatine storage capacity. When phosphocreatine reserves in skeletal muscle are depleted, glucose, glycogen, and fatty acids are oxidized [5]. However, during the growing phase, fatty acid reserves can be very low [6], especially in zebu breeds [7], making carbohydrates and the phosphocreatine/creatine system the primary energy sources for muscle growth [8].

Guanidinoacetic acid (GAA) is an intermediate metabolite synthesized from L-arginine and glycine in the kidneys and transported through the bloodstream to the liver, where it plays a precursor role in creatine biosynthesis [9,10,11,12], essential for muscle energy metabolism [13]. Creatine is a key metabolite for mitochondrial ATP synthesis and protein synthesis via the mTOR pathway, enhancing the phosphorylation of p70S6K, which stimulates muscle hypertrophy [14,15]. Additionally, creatine increases intramuscular phosphocreatine levels, improving ATP production and supporting skeletal muscle growth [14,15].

Several efforts have been made to explore the use of GAA as a feed additive to serve as an alternative source of creatine for meat-producing farm animals. Studies conducted with poultry and swine supplemented with GAA have shown that GAA can stimulate protein synthesis and muscle growth, improving weight gain, feed efficiency, and meat quality [16,17,18,19]. In beef cattle, it was recently demonstrated that the addition of 0.6 g of GAA per kg of dry matter resulted in increased weight gain and improved feed efficiency in finishing diets for taurine breed bulls [20,21,22].

However, studies investigating the inclusion of GAA in the supplementation of *Bos taurus indicus* cattle in the pasture rearing or feedlot finishing phase, especially in tropical conditions, still need to be explored. The hypothesis was that feeding increasing levels of GAA would enhance energy metabolism and protein synthesis, thereby improving performance during the growing and finishing phases, carcass traits, and meat quality in Nellore bulls. Thus, this study was carried out to evaluate the effect of increasing levels of dietary GAA on performance during the growing and finishing phases, as well as on the carcass characteristics and meat quality of young Nellore bulls.

## 2. Materials and Methods

The feeding management and data collection for the animals in this study were conducted at the School of Animal Science and Food Engineering of University of São Paulo Pirassununga/SP, Brazil. The Ethics Committee approved the study on the Use of Animals under protocol CEUA nº 9222010623.

### 2.1. Animals, Management, Diet, and Experimental Design

Fifty-two newly weaned Nellore bulls (initial body weight [BW] of 265 ± 19 kg and age of 8 ± 2 months) were blocked by the initial BW (*n* = three blocks) and randomly assigned to one of the following treatments: Control, no added GAA (CON, *n* = 13); addition of 3 g/kg DM GAA (GAA3, *n* = 13); addition of 6 g/kg DM GAA (GAA6, *n* = 13); and addition of 9 g/kg DM GAA (GAA9, *n* = 13). During the growing phase, the bulls received a protein-energy supplement at a rate of 3 g/kg of BW (Table 1), adjusted to supply 3, 6, and 9 g of GAA per kg of DM, according to the respective treatments. During the finishing phase in the feedlot, GAA was included in the concentrate and subsequently provided in the total mixed ration (TMR) at reduced concentrations of 0.3, 0.6, and 0.9 g/kg DM GAA per kg of DM, respectively. This reduction reflected the greater dry matter intake during finishing compared to the supplement provided during the growing phase. The experimental design was such that the total target intake of GAA remained equivalent between phases, regardless of the feeding method.

The growing phase lasted 280 days and was conducted on approximately 25.6 hectares of *Urochloa decumbens* pasture, divided into 16 paddocks of 1.6 hectares each. Twelve paddocks were used for the experiment, and they were equipped with waterers and feed troughs for supplement provision. Each treatment was assigned to three paddocks, with groups of four or five bulls per paddock, and each paddock was considered an experimental unit. During this period, bulls were supplemented daily at 8:00 a.m., ensuring a minimum of 30 cm of trough space per bull, regardless of the number of bulls in the paddock, along with free access to water. The amount of supplement offered was adjusted every 28 days based on bull BW to ensure proper supplement provision.

The height and forage mass of each paddock were measured every 56 d to calculate the available forage mass. Forage height was measured using a measuring tape attached to a wooden rod, with 50 measurements per paddock taken in a zigzag pattern, as described by Barioni and Ferreira [23]. Forage mass was determined using a 0.25 m^2^ square with cuts close to the ground, with samples collected from five points: one at each extremity and one in the center of the paddock [23]. Based on the forage mass assessments, each experimental unit was rotated among the paddocks in a controlled manner to ensure similar pasture availability across treatments and minimize discrepancies. Additionally, four paddocks were reserved and, when used, were equally distributed among the experimental blocks.

At the beginning of the growing phase, the bulls were subjected to a health protocol, including vaccination against clostridial diseases (Poli-Star^®^, MSD Saúde Animal, Cruzeiro, SP, Brazil) and deworming with Levamisole (Ripercol L^®^ 150F, Zoetis, Campinas, SP, Brazil), each administered once according to the manufacturer’s recommendations. At the end of the growing phase, one bull from the GAA6 treatment group suffered a bone injury and was removed from the study.

After the growing phase, the bulls from each experimental unit were weighed and reorganized according to BW within their respective treatments to form blocks for finishing in the feedlot. Four or five bulls from each treatment were allocated to separate collective pens, resulting in 17 bulls per pen across three pens. Each pen was equipped with four Intergado^®^ electronic feeders (Betim, MG, Brazil), capable of measuring individual feed intake. Each feeder was fitted with electronic gates that allowed access only to specific bulls, restricting access to other animals not assigned to that feeder’s treatment. This setup ensured that all treatment diets were represented within each pen, with bulls from different treatments housed together but consuming only their designated diets (Table 2). Additionally, each pen featured a drinking trough integrated with an electronic scale from Intergado^®^ to monitor the BW of the animals. During the finishing phase, each bull was considered an experimental unit. The total feedlot period lasted 94 days, including 20 days of adaptation to the facilities and the finishing diet. A “step-up” adaptation protocol was used, transitioning the forage-to-concentrate ratio as follows: 100:0, 75:25, 50:50, and 25:75, with each step lasting five days, using corn silage as the forage source. The final diet was provided once daily at 8:00 a.m. Feed was offered based on the amount of orts from the previous day, and in cases of excessive orts above 5% of the amount offered, they were removed, and the diet was adjusted.

### 2.2. Pasture and Ingredient Sampling and Nutritional Composition Analysis

Pasture samples from each paddock were collected every 56 days, one week before the rotation of the animals between paddocks. Subsequently, a composite sample was prepared for each treatment. Regarding the supplement for the growing phase, samples of the ingredients were collected with each new batch of concentrate (produced every 60 days) and stored at −20 °C for further analysis. During the finishing phase in feedlot, samples of corn silage and concentrate ingredients were collected weekly, and composite samples were subsequently formed for both the silage and each ingredient, covering the entire feedlot period. All samples were stored at −20 °C until the nutritional profile analysis (Table 3 and Table 4). Before analysis, the samples were thawed and dried in a forced-ventilation oven at 55 °C for 96 h, following the methodology described by AOAC ([24], method #930.15). Subsequently, the samples were ground through a 1 mm mesh sieve using a Willey knife mill (Marconi, Pirassununga, SP, Brazil).

The dry matter content was determined by drying the samples in an oven at 105 °C for 24 h ([24], method #934.01). The ash content was determined by incinerating the samples in a muffle furnace at 550 °C for 4 h [24]. The concentration of total nitrogen (N) was determined by the Dumas method using the LECO FP-528 (Leco Corporation, St. Joseph, MI, EUA; ([27], method #990.03)). Crude protein (CP) was calculated by multiplying the total N content by 6.25. The neutral detergent fiber (NDF) and acid detergent fiber (ADF) contents were determined according to the Van Soest method [28,29], using thermostable alpha-amylase and sodium sulfite. The analyses were performed using the classical crucible method, and the NDF and ADF values obtained were subsequently corrected for ash. Hemicellulose was calculated based on the difference between the NDF and ADF values [28]. The ether extract (EE) content was determined using a soxhlet system with a paper filter and petroleum ether ([24], method #920.39). The total digestible nutrients (TDN) of pasture, supplement, corn silage and ingredients were calculated according to Weiss et al. [25]. Net energy for maintenance and net energy from gain were tabulated and calculated according to NASEM [26].

### 2.3. Performance and Carcass Ultrasound

The bulls’ BW during the growing phase was recorded every 28 days, without fasting, after supplements were provided to adjust the supplement supply and monitor weight gain. Average daily gain (ADG) was calculated based on the difference between the final BW and initial BW during the growing period and divided by the number of days in the growing period.

During the finishing phase, after 20 days of adaptation to the facilities and feedlot diet, the initial and final BW, as well as the ADG, were estimated based on the weights recorded daily without fasting using the Intergado^®^ electronic scale system, according to the regression model proposed by Archer and Bergh [30]. Dry matter intake (DMI) was obtained from the individual daily records of the Intergado^®^ electronic feeders, which provided the data on an as-fed basis. Using this information and the diet’s dry matter content, the average DMI over the experimental period was calculated. The percentage of DMI relative to BW was calculated by dividing the DMI by BW and multiplying the result by 100. Feed efficiency (FE) during the finishing phase was calculated by dividing the ADG by DMI, expressed as kg of weight gain per kg of dry matter intake. Water consumption was measured based on daily records from Intergado^®^ electronic water fountains.

Carcass evaluation by ultrasound was performed every 56 days during the growing phase and at the beginning, middle, and end of the experimental finishing period in the feedlot. Ribeye area (REAu) and backfat thickness (BFTu) measurements were taken on the *Longissimus thoracis* muscle between the 12th and 13th thoracic vertebrae, and rump fat thickness (RFTu) in the Biceps femoris muscle between the ileum and ischium [31], using an Exago device (IMV do Brasil, Campinas, Brazil), with a 180 mm, 3.5 mHz linear array transducer. All ultrasound images collected were analyzed using Lince^®^ V.15 software (M&S Consultoria Agropecuária Ltd.a, Pirassununga, Brazil).

### 2.4. Bull Slaughter and Carcass Traits

The slaughter was carried out at the University of Sao Paulo abattoir, located approximately 300 m from the experimental feedlot. The animals were slaughtered in four groups over a 14-day period, with each group formed based on BFTu and an equal number of animals per treatment.

Sixteen hours before slaughter, the animals were subjected to total feed restriction, with free access to water. Before loading into trucks their shrunk BW was recorded. Animals were transported by truck in the morning of slaughter. All slaughter procedures were conducted humanely according to the Regulations of the Industrial and Sanitary Inspection of Products of Animal Origin [32]. During slaughter, after the removal of the head, legs, hide, and viscera (including kidneys, diaphragm and visceral fat) the hot carcass weight (HCW) was recorded and the carcass was subsequently moved to a cooler maintained at 4 °C. After 24 h of chilling the carcasses were weighed again to determine the cold carcass weight (CCW). Carcass yield (CY), in percentage, was calculated by dividing the HCW by the fasting BW and multiplying the result by 100.

After 24 h of refrigeration, the primal cuts of the left half carcass were separated into the forequarter, thin flank, and hindquarter. The forequarters were separated between the 5th and 6th thoracic vertebrae, while the hindquarters were separated from the flank with a cut between the 12th and 13th thoracic vertebrae. The yield of each primal cut was calculated as a percentage by dividing the weight of each primal cut by the HCW and multiplying the result by 100.

The carcass pH and temperature were measured in the *Longissimus thoracis* muscle, between the 11th and 12th thoracic vertebrae, at 1 and 24 h after slaughter. Measurements were performed using a pH meter equipped with a penetration probe (model HI99163, Hanna Instruments, São Paulo, SP, Brazil) and a digital thermometer (Incoterm, Porto Alegre, RS, Brazil).

### 2.5. Meat Quality Sample Collection and Analysis

The meat samples were collected 24 h *post-mortem*. A total of six steaks (2.5 cm thick) of the *Longissimus thoracis* muscle between the 10th and 12th thoracic vertebrae were collected from the left side of each animal. Four steaks were used for the analysis of color, cooking loss (CL), and Warner-Bratzler shear force (WBSF) at four different aging times (1, 7, 14, and 21 days). Among these four steaks, one was immediately analyzed for color, CL, and FC, while the other three steaks were individually vacuum-packed and refrigerator-aged (2 °C) for 7, 14, and 21 days, respectively, for later analysis.

Color evaluation was performed by removing the samples from the vacuum packaging and exposing them to air for 30 min to bloom under cold conditions. After blooming, the surface color of the steaks was analyzed at three different positions using a CM2500d spectrophotometric colorimeter (Konica Minolta Brasil, São Paulo, SP, Brazil). The equipment was configured with a 10 mm aperture, calibrated with illuminant A and an observation angle of 10°, allowing the L*, a*, and b* values to be obtained [33].

The same steaks used for color evaluation were subsequently used to determine CL and WBSF, following the protocol established by the American Meat Science Association [34]. After blooming, the steaks were kept at room temperature and weighed to record their initial weight. A thermometer was inserted into the geometric center of each sample, and the steaks were cooked in an industrial electric oven (model F130/L, Fornos Elétricos Flecha de Ouro Ind. e Com. Ltd.a., São Paulo, SP, Brazil) at 170 °C until the internal temperature reached 40 °C. The samples were turned over and kept in the oven until they reached 71 °C. After removal from the oven, the samples were left to cool at room temperature (25 °C) and weighed again. Cooking loss was expressed as a percentage using the equation: CL (%) = [(Initial weight − Final weight)/Initial weight] × 100. Subsequently, the samples were wrapped in plastic film and stored under refrigeration (4 to 6 °C) overnight (approximately 12 h). After refrigeration, six cylindrical cores (1.27 cm diameter) were removed from each steak, parallel to the orientation of the muscle fibers, using a bench drill. Shear force was measured using a Warner-Bratzler blade attached to a texture analyzer (Model TMSPRO, Food Technology Corporation, Sterling, VA, USA) at a 200 mm/min crosshead speed. The Warner-Bratzler shear force (WBSF) was determined as the average of the peak force values obtained from the six cores, and the results were expressed in Newtons (N).

The fifth steak was collected for sarcomere length and chemical composition analysis. For sarcomere length analysis, five smaller pieces, weighing approximately one gram each, were sampled from each steak at different anatomical positions to represent the entire surface area, wrapped in aluminum foil, and frozen in liquid nitrogen. The remainder of the fifth steak had the subcutaneous fat removed and was ground for analysis of chemical composition via near-infrared analysis using a FoodScanTM (FOSS, Hillerod, Denmark; ([35], method: 2007-04)). The sixth and last steak was analyzed for myofibrillar fragmentation index (MFI). The steak was cut into four pieces for this analysis and pieces were randomly selected to be subject to different aging times. The piece for the first aging time (1 day) was wrapped in aluminum foil and immediately frozen in liquid nitrogen, while the remaining pieces were vacuum packed and aged (2 °C) for 7, 14, and 21 days, respectively. After each target aging time, the samples were removed from the vacuum package, cut, wrapped in aluminum foil and frozen in liquid nitrogen.

Myofibrillar fragmentation index (MFI) analysis was performed according to Culler et al. [36], with a modification in the extraction process described by Ramos et al. [37]. Sarcomere length was determined by measuring 32 sarcomeres per animal, following the methodology described by Cross et al. [38] and Koolmees et al. [39]. The results were expressed in micrometers (µm).

### 2.6. Statistical Analysis

The data were analyzed by analysis of variance, with treatments considered fixed effects and block (initial BW block) as random. For variables evaluated as repeated measures over time, such as carcass ultrasound (REAu, BFTu, and RFTu) and meat quality after aging (CL, color, WBSF, and MFI), a first-order autoregressive structure was used, with time specified as the repeated factor within each experimental unit, the bull considered as the subject, and block as a random effect. Final BW was obtained from the regression of daily individual weights recorded by the Intergado^®^ system during the finishing phase. DMI was calculated as the total feed intake per bull divided by the number of days, resulting in an average DMI value per animal. For single-measure variables, such as initial and final BW, ADG, DMI, and FE, water intake, as well as carcass traits such as HCW, CCW, CY, primal cuts, REA, BFT, pH, temperature, and meat quality (chemical composition and sarcomere length), the model included treatment as a fixed effect and block as a random effect. During the growing phase, the paddock was considered the experimental unit, whereas during the finishing phase, the animal was considered the experimental unit.

Polynomial contrasts were employed to evaluate linear, quadratic, and deviation trends of treatments on the dependent variables. Adjusted means were calculated using the LSMEANS statement, while trends were evaluated with the CONTRAST command. Statistical analysis was performed using SAS software (SAS 9.4 Inst., Inc., Cary, NC, USA, 2013) with the MIXED procedure. Residual normality was assessed using the Shapiro-Wilk test, and homogeneity of variances was tested with Levene’s method. Outliers were removed, when necessary, based on the r-value from the student’s test. Effects were considered significant when *p* ≤ 0.05, and trends were reported for 0.05 < *p* < 0.10.

## 3. Results

The GAA supplementation did not affect the bulls’ BW or ADG during the growing phase (*p* ≥ 0.152; Table 5). Similarly, for carcass ultrasound measurements (REAu, BFTu, and RFTu), no interaction between time and treatment was observed (*p* ≥ 0.771), and no effect of GAA supplementation was detected (*p* ≥ 0.117; Table 6). However, regardless of treatment, REAu, BFTu, and RFTu values increased over time during the growing period (*p* < 0.001; Table S1).

During the finishing phase, GAA supplementation did not affect performance variables or feed and water intake (*p* ≥ 0.107; Table 7).

There was no interaction between treatment and time during the feedlot phase for carcass ultrasound variables such as REAu, BFTu, and RFTu (*p* ≥ 0.437; Table 8). The GAA supplementation also did not affect these variables (*p* ≥ 0.115). However, as expected, differences in carcass ultrasound measurements were observed over time, with increased values as the feedlot period progressed (*p* < 0.001; Table S2). Regarding carcass characteristics after slaughter, GAA supplementation did not affect any of the variables analyzed (*p* ≥ 0.100; Table 9).

The chemical composition of the meat was not affected by GAA supplementation (*p* ≥ 0.113; Table 10). Regarding meat quality traits, no interaction between treatment and aging time was observed for any variable (*p* ≥ 0.712), nor was there any effect of GAA supplementation alone (Table 11). Aging time significantly affected all meat quality variables (*p* < 0.001; Table S3) except for L* values (*p* = 0.273). As aging progressed, the values of a*, b*, and MFI increased while CL and WBSF decreased. Additionally, a linear decreasing effect was observed for CL (*p* = 0.015), with lower values in the GAA9 group than CON.

## 4. Discussion

In tropical beef cattle production, the growing phase is challenging, where newly weaned calves are managed on pastures during the dry season. Seasonality affects forage availability and quality, making the growing phase the longest in the production cycle [40]. Thus, feeding strategies such as supplementation and additives have become common practices to prevent growth losses and shorten the time required to reach the finishing phase [41], including rumen modulators and, more recently, metabolic regulators such as GAA, which target energy and protein metabolism [42].

Guanidinoacetic acid (GAA) aims to positively influence muscle. Guanidinoacetic acid is a natural precursor of creatine synthesized in the liver of animals [43], and 70% of creatine needs are met by endogenous synthesis [44]. After its formation, creatine is released by the liver into the circulation and taken up by various tissues, including skeletal muscle, through specific membrane transporters such as SLC6A8 [45,46]. In muscle tissue, creatine is phosphorylated by the enzyme creatine kinase to form phosphocreatine, a molecule that serves as an energy source during ATP depletion [13]. It is worth mentioning that creatine is absent in plant organisms and can only be found in animal by-products [47]. However, adding animal by-products to ruminant diets is prohibited [48]. Thus, GAA supplementation may be an appropriate alternative to increase creatine reserves in ruminants [47].

Although skeletal muscle is one of the main targets of GAA in energy metabolism, feeding unprotected GAA to ruminants may be problematic because it may be utilized by ruminal microorganisms. Previous studies estimated that approximately 51% of the dietary GAA could be degraded or utilized by the ruminal microbiota [49]. Furthermore, increasing GAA doses (0.3, 0.6, and 0.9 g/kg DM) had a linear effect on the populations of fibrous carbohydrate-fermenting microorganisms, increasing the digestibility of neutral and acid detergent fiber as well as the total concentration of short-chain fatty acids in the rumen [20].

In the present study, although the digestibility of forage consumed by young bulls was not evaluated, adequate fiber digestion was expected to result in better performance of GAA-supplemented animals [20] on pasture compared to the CON group. However, in addition to the absence of differences in final BW during the growth phase, GAA supplementation did not affect REAu measurements related to carcass musculature. These results suggest that the dose of GAA may not have been sufficient to reach its target, skeletal muscle, to stimulate protein synthesis as previously found in non-ruminant organisms.

Guanidinoacetic acid degradation in the rumen, which was not assessed in the present study, may be greater under grazing conditions, exceeding the 51% reported by Speer et al. [49], due to the nitrogen deficiency characteristic of tropical pastures during the dry season [2]. In the present study, the pasture contained 5.20% crude protein, and despite supplementation, the average daily protein intake from the supplement was estimated at approximately 330 g. To avoid replacing pasture with concentrated feed, a common practice in tropical systems, a low-level supplementation (3 g per kg of body weight) was chosen. Higher levels of supplementation may disrupt the balance between protein and energy in tropical pastures, particularly during the rainy season [3]. Therefore, the objective was to promote animal growth while maintaining the forage as the primary nutrient source [3]. It is known that the ruminal microbiota efficiently utilizes small nitrogen- and hydrogen-rich molecules. Thus, GAA, a small molecule (117.11 g/mol) composed of three nitrogen atoms and mainly in its radicals [50], could be used by bacteria that rely exclusively on non-protein nitrogen for microbial protein synthesis, such as fiber-degrading bacteria [51,52]. In ruminants fed with forages, the ruminal escape of nitrogen is low, with microbial protein being the primary source of available nitrogen [52].

In contrast, Ardalan et al. [53] reported that under methionine-deficient conditions, GAA supplementation does not contribute to nitrogen retention or body growth in cattle, as creatine synthesis in the liver requires methyl group donation. Muscle cells primarily absorb synthetic creatine [54], which is used to permanently grow skeletal muscle [55]. However, creatine synthesis is a process that demands high levels of methyl groups, and GAA supplementation can increase this demand [56,57]. In tropical conditions, methionine is often the limiting amino acid for cattle growth [58,59]. As highlighted in the review by Crouse et al. [60], one-carbon metabolism plays a key role in nitrogen retention and protein digestion efficiency, both essential processes for animal growth. In this context, a methionine deficiency in the diet may limit the availability of methyl groups required for converting GAA to creatine, thereby impairing the expected effects of GAA on muscle metabolism. This limitation may help explain the lack of performance differences between GAA-supplemented bulls and the CON group during the pasture-based growing phase. Future studies evaluating the combined supplementation of GAA and methionine may be crucial to better understand the influence of methyl group availability on energy metabolism and muscle growth in cattle.

Previous studies highlighted the positive effect of GAA supplementation between 0.6 and 1.6 g/kg DM in beef cattle, with additional ADG increases ranging from 0.14 to 0.39 kg/day [20,21,47,61]. In contrast, Eckhardt et al. [62], in a study with 146 days in the feedlot, found no differences in ADG in Angus bulls supplemented with 1 to 2 g of GAA per 100 kg body weight compared to an unsupplemented group, which coincides with our results, as we also found no differences in these performance variables during the finishing phase. Differences in feedlot duration, diet energy density, and breed may explain the variations in results between studies. Carvalho et al. [63] observed performance differences between Angus and Nellore cattle, with Angus showing greater nutrient intake and digestibility in diets with a high grain content during the finishing phase. These differences in intake capacity and digestibility likely influence the response to GAA supplementation, contributing to the variations in performance results observed in studies with Angus [20,21,47,61], compared with the results obtained in the present study with Nellore.

Guanidinoacetic acid supplementation also creates divergent effects on DMI in the finishing phase. While some studies report increased DMI due to GAA supplementation [20], others found no significant differences [22,47,62], and, in some cases, reported reduced DMI [49]. However, in the present study, no differences in DMI were observed during the finishing phase as a result of GAA inclusion in the diet.

Several studies have investigated the effect of GAA on meat composition and quality in ruminants, showing varied results for different measured traits. Some research indicates that GAA can influence parameters such as color [61,64], pH [65,66], meat chemical composition [65,66], shear force and cooking loss [64,65,66]. In contrast, some studies found no effects of GAA on color [62,65,66], pH [61,62], meat chemical composition [62], shear force [61,62,64,65] and cooking loss [61,62].

In the present study, the only observed effect on meat quality parameters was cooking loss, which was lower in the GAA9 group compared to the other treatments during the feedlot. Greater water retention in skeletal muscle may be associated with increased creatine concentration in the tissue due to GAA intake [17,61,66]. However, as creatine concentration was not measured in the present study, this remains a hypothesis based on previous findings. Further studies quantifying intramuscular creatine content are needed to confirm this potential mechanism. Creatine is a water-soluble and highly polar molecule that regulates cellular osmosis [67]. Thus, in a scenario of greater energy bioavailability, osmotic regulation becomes essential for protein synthesis [68]. Water exists in three forms within the skeletal muscle structure: free, immobilized, and bound, and variations among these forms influence the amount of water that can be lost in meat [69]. In muscle, the portion of water that remains associated with muscle proteins through electrostatic interactions is called bound water. Both bound water and muscle proteins play a key role in retaining immobilized water within the muscle [69,70]. Therefore, if GAA did indeed increase retention of these more internal water fractions in the muscle, the result may have been the observed reduction in cooking losses.

## 5. Conclusions

Increasing doses of GAA supplementation did not improve the performance of Nellore bulls during the growing phase on pasture. Similarly, GAA supplementation did not affect performance during the feedlot finishing phase. Carcass characteristics were also not influenced by GAA supplementation. In contrast, there was a linear reduction in cooking losses with increasing GAA doses. Based on these results, future studies are warranted to evaluate the use of rumen-protected GAA, alone or in combination with rumen-protected methionine, in grazing and feedlot systems to enhance performance, carcass characteristics, and meat quality potentially.

## Figures and Tables

**Table 1:** Composition of the supplement used during the growing phase.

Ingredients (g/kg)	Growing Supplement ^1^			
	CON	GAA3	GAA6	GAA9
Ground corn	584	581	578	575
Soybean meal 45%	350	350	350	350
Urea	25.0	25.0	25.0	25.0
Sodium chloride	20.0	20.0	20.0	20.0
Mineral premix ^2^	21.0	21.0	21.0	21.0
Guanidinoacetic Acid	-	3.0	6.0	9.0

^1^ CON: Without adding guanidinoacetic acid (GAA); GAA3: Addition of 3 g/kg of DM GAA; GAA6: Addition of 6 g/kg of DM GAA; GAA9: Addition of 9 g/kg of DM GAA. ^2^ Minerthal 160 MD composition: Ca = 230 g/kg, P = 160 g/kg, S = 60 g/kg, Co = 160 mg/kg, Cu = 270 mg/kg, F = 1600 mg/kg, I = 135 mg/kg, Mn = 2700 mg/kg, Se = 80 mg/kg, Zn = 8100 mg/kg, Sodium Monensin = 4000 mg/kg.

**Table 2:** Composition of the experimental diets used during the finishing phase.

Ingredients (g/kg)	Finishing Diet ^1^			
	CON	GAA3	GAA6	GAA9
Corn silage	248.0	248.0	248.0	248.0
Ground corn	648.9	648.6	648.3	647.9
Soybean meal 45%	69.6	69.6	69.6	69.6
Urea	11.6	11.6	11.6	11.6
Mineral premix ^2^	20.0	20.0	20.0	20.0
Potassium chloride	1.5	1.5	1.5	1.5
Magnesium oxide ^3^	0.3	0.3	0.3	0.3
Guanidinoacetic Acid	-	0.3	0.6	0.9

^1^ CON: No added guanidinoacetic acid (GAA); GAA3: Addition of 0.3 g/kg of DM GAA; GAA6: Addition of 0.6 g/kg of DM GAA; GAA9: Addition of 0.9 g/kg of DM GAA. ^2^ GuabiTech feedlot premix composition: Ca = 220 g/kg; P = 20 g/kg; Na = 60 g/kg; S = 30 g/kg; Mg = 20 g/kg; K = 25 g/kg; Co = 13.30 g/kg; Cu = 446.2 mg/kg; I = 28.3 mg/kg; Mn = 490 mg/kg; Se = 5.37 mg/kg; Zn = 1332 mg/kg; Cr = 20 mg/kg; F = 200 mg/kg; Mannanoligosaccharide = 1511.4 mg/kg; *Saccharomyces cerevisae* = 2.25 × 10^10^ CFU/kg; Sodium Monensin = 1.155 mg/kg; Beta-glucans = 4732 mg/kg. ^3^ Phix-up: magnesium oxide (Min)= 470 g/kg.

**Table 3:** Nutritional composition of *Urochloa decumbens* pasture and the supplement used during the growing phase.

Item ^1^	*Urochloa decumbens*	Supplement
Dry matter, %	37.5	89.3
Crude protein, % DM	5.2	31.0
Neutral detergent fiber, % DM	74.0	9.9
Acid detergent fiber, % DM	41.0	3.8
Hemicellulose, % DM	33.0	6.0
Ether extract, % DM	1.6	2.7
Ash, % DM	7.1	6.0
Non-fibrous carbohydrate, % DM	12.1	50.4
Total digestible nutrients ^2^	31.2	80.9
Net energy for maintenance ^3^, Mcal/kg	0.3	2.0
Net energy gain ^3^, Mcal/kg	0.1	1.3

^1^ Nutritional profile of each ingredient was analyzed using wet chemistry procedures [24]. DM: dry matter. ^2^ Calculations were performed according to the equations proposed by Weiss et al. [25]. ^3^ Composition calculated using tabular values from NASEM [26].

**Table 4:** Nutritional composition of corn silage and concentrate used during the finishing phase.

Item ^1^	Corn Silage	Concentrate
Dry matter, %	30.0	87.8
Crude protein, % DM	9.3	17.0
Neutral detergent fiber, % DM	43.7	9.6
Acid detergent fiber, % DM	25.4	2.8
Hemicellulose, % DM	18.3	6.8
Ether extract, % DM	3.0	4.3
Ash, % DM	4.3	4.1
Non-fibrous carbohydrate, % DM	39.7	65.3
Total digestible nutrients ^2^	71.0	74.2
Net energy for maintenance ^3^, Mcal/kg	1.7	1.8
Net energy gain ^3^, Mcal/kg	1.1	1.1

^1^ Nutritional profile of each ingredient was analyzed using wet chemistry procedures [24]. DM: dry matter. ^2^ Calculations were performed according to the equations proposed by Weiss et al. [25]. ^3^ Composition calculated using tabular values from NASEM [26].

**Table 5:** Effect of supplementation with or without GAA on the performance of bulls during the growing phase.

Item ^1^	Treatments ^2^				SEM ^3^	*p*-Value ^4^		
	CON	GAA3	GAA6	GAA9		L	Q	Deviation
Initial BW, kg	269.4	269.5	269.2	270.0	3.10	0.915	0.915	0.924
Final BW, kg	513.4	495.2	496.2	500.4	8.31	0.316	0.192	0.654
ADG, kg/d	0.81	0.77	0.76	0.76	50.0	0.152	0.490	0.980

^1^ Initial BW: initial body weight; Final BW: final body weight; ADG: average daily gain. ^2^ CON: No added guanidinoacetic acid (GAA); GAA3: Addition of 3 g/kg of DM GAA; GAA6: Addition of 6 g/kg of DM GAA; GAA9: Addition of 9 g/kg of DM GAA. ^3^ SEM: Standard error of the mean. ^4^ L: linear effect; Q: quadratic effect; Deviation: Quadratic deviation.

**Table 6:** Effect of supplementation with or without GAA on carcass measurements by ultrasound of bulls during the growing phase.

Item ^1^	Treatments ^2^				SEM ^3^	*p*-Value ^4^				
	CON	GAA3	GAA6	GAA9		L	Q	Deviation	Time	T × T
REAu, cm^2^	54.6	53.6	52.0	55.1	1.29	0.977	0.117	0.360	<0.001	0.960
BFTu, mm	1.1	1.3	1.3	1.3	0.22	0.669	0.812	0.635	<0.001	0.771
RFTu, mm	2.5	2.6	2.5	2.3	0.36	0.594	0.707	0.964	<0.001	0.982

^1^ REAu: ultrasound ribeye area; BFTu: ultrasound backfat thickness; RFTu: ultrasound rump fat thickness. ^2^ CON: No added guanidinoacetic acid (GAA); GAA3: Addition of 3 g/kg of DM GAA; GAA6: Addition of 6 g/kg of DM GAA; GAA9: Addition of 9 g/kg of DM GAA. ^3^ SEM: Standard error of the mean. ^4^ L: linear effect; Q: quadratic effect; Deviation: Quadratic deviation; T × T: treatment × time interaction.

**Table 7:** Performance of Nellore bulls fed increasing doses of GAA during the finishing phase.

Item ^1^	Treatments ^2^				SEM ^3^	*p*-Value ^4^		
	CON	GAA3	GAA6	GAA9		L	Q	Deviation
Initial BW, kg	506.6	498.6	490.9	493.6	10.7	0.326	0.618	0.839
Final BW, kg	631.2	630.1	609.4	610.6	13.8	0.186	0.934	0.512
ADG, kg/d	1.6	1.7	1.6	1.6	0.09	0.315	0.503	0.367
DMI, kg/d	10.5	10.3	9.6	9.9	0.38	0.162	0.503	0.399
DMI, %BW	1.7	1.7	1.9	1.9	0.08	0.107	0.557	0.430
FE, kg ADG/kg DM	0.16	0.17	0.17	0.16	0.01	0.799	0.177	0.779
Water intake, L/d	34.8	33.7	33.7	31.9	1.90	0.312	0.873	0.733

^1^ Initial BW: initial body weight; Final BW: final body weight; ADG: average daily gain; DMI: dry matter intake; FE: feed efficiency. ^2^ CON: No added guanidinoacetic acid (GAA); GAA3: Addition of 0.3 g/kg of DM GAA; GAA6: Addition of 0.6 g/kg of DM GAA; GAA9: Addition of 0.9 g/kg of DM GAA. ^3^ SEM: Standard error of the mean. ^4^ L: linear effect; Q: quadratic effect; Deviation: Quadratic deviation.

**Table 8:** Effect of supplementation with increasing doses of GAA on carcass ultrasound measurements of Nellore bulls during the finishing phase.

Item ^1^	Treatments ^2^				SEM ^3^	*p*-Value ^4^				
	CON	GAA3	GAA6	GAA9		L	Q	Deviation	Time	T × T
REAu, cm^2^	78.7	78.0	78.0	79.1	0.85	0.732	0.280	0.927	<0.001	0.437
BFTu, mm	2.6	2.6	2.6	2.6	0.02	0.640	0.953	0.118	<0.001	0.881
RFTu, mm	6.3	6.3	5.4	6.1	0.33	0.269	0.291	0.115	<0.001	0.999

^1^ REAu: ultrasound ribeye area; BFTu: ultrasound backfat thickness; RFTu: ultrasound rump fat thickness. ^2^ CON: No added guanidinoacetic acid (GAA); GAA3: Addition of 0.3 g/kg of DM GAA; GAA6: Addition of 0.6 g/kg of DM GAA; GAA9: Addition of 0.9 g/kg of DM GAA. ^3^ SEM: Standard error of the mean. ^4^ L: linear effect; Q: quadratic effect; Deviation: Quadratic deviation; T × T: treatment × time interaction.

**Table 9:** Effect of supplementation with increasing doses of GAA on carcass traits of Nellore bulls post-slaughter.

Item	Treatments ^1^				SEM ^2^	*p*-Value ^3^		
	CON	GAA3	GAA6	GAA9		L	Q	Deviation
Shrunk body weight, kg	624.1	621.1	606.0	607.9	12.4	0.245	0.847	0.620
Hot carcass weight, kg	362.7	354.7	348.7	348.4	7.10	0.122	0.595	0.913
Cold carcass weight, kg	357.3	349.2	343.2	343.5	7.16	0.135	0.566	0.901
Carcass yield, %	58.1	57.1	57.5	57.3	0.27	0.149	0.191	0.168
Forequarter, kg	142.5	137.0	137.5	135.2	2.92	0.100	0.584	0.518
Thin flank, kg	52.5	52.1	49.6	50.2	1.55	0.178	0.740	0.492
Hindquarter, kg	162.3	160.2	156.1	158.5	3.32	0.251	0.547	0.608
Forequarter yield, %	22.5	21.7	21.7	21.4	0.45	0.101	0.584	0.518
Thin flank yield, %	8.3	8.24	7.85	8.0	0.24	0.180	0.746	0.492
Hindquarter yield, %	26.0	25.8	25.8	26.0	0.22	0.940	0.392	0.948
Initial pH	6.6	6.8	6.8	6.7	0.09	0.383	0.147	0.897
Final pH (24 h)	5.6	5.6	5.6	5.7	0.04	0.214	0.588	0.264
Initial temperature, °C	40.4	40.2	40.0	40.2	0.17	0.405	0.300	0.668
Final temperature (24 h), °C	6.3	6.1	5.8	6.2	0.24	0.557	0.192	0.607

^1^ CON: No added guanidinoacetic acid (GAA); GAA3: Addition of 0.3 g/kg of DM GAA; GAA6: Addition of 0.6 g/kg of DM GAA; GAA9: Addition of 0.9 g/kg of DM GAA. ^2^ SEM: Standard error of the mean. ^3^ L: linear effect; Q: quadratic effect; Deviation: Quadratic deviation.

**Table 10:** Effect of supplementation with increasing doses of GAA on the proximate chemical composition of meat from Nellore bulls.

Item, %	Treatments ^1^				SEM ^2^	*p*-Value ^3^		
	CON	GAA3	GAA6	GAA9		L	Q	Deviation
Moisture	72.3	72.1	72.4	72.5	0.21	0.431	0.592	0.371
Proteins	23.2	23.2	23.2	23.4	0.14	0.520	0.554	0.601
Minerals	3.0	3.0	3.1	3.0	0.14	0.997	0.749	0.661
Collagen	1.7	1.7	1.5	1.7	0.06	0.869	0.143	0.113
Lipids	1.4	1.3	1.3	1.2	0.13	0.218	0.732	0.654

^1^ CON: No added guanidinoacetic acid (GAA); GAA3: Addition of 0.3 g/kg of DM GAA; GAA6: Addition of 0.6 g/kg of DM GAA; GAA9: Addition of 0.9 g/kg of DM GAA. ^2^ SEM: Standard error of the mean. ^3^ L: linear effect; Q: quadratic effect; Deviation: Quadratic deviation.

**Table 11:** Effect of supplementation with increasing doses of GAA on the meat quality of Nellore bulls.

Item	Treatments ^1^				SEM ^2^	*p*-Value ^3^				
	CON	GAA3	GAA6	GAA9		L	Q	Deviation	Time	T × T
Color										
Lightness (L*)	37.5	37.1	36.9	36.7	0.40	0.153	0.755	0.900	0.273	0.832
Redness (a*)	22.8	22.7	22.7	23.2	0.36	0.370	0.869	0.869	<0.001	0.665
Yellowness (b*)	15.7	15.2	15.0	15.8	0.41	0.974	0.129	0.612	<0.001	0.712
Cooking loss, %	28.0	27.6	27.1	27.1	0.28	0.015	0.512	0.501	<0.001	0.887
WBSF ^4^, N	57.6	56.7	58.5	58.8	1.10	0.260	0.563	0.412	<0.001	0.746
Myofibrillar Fragmentation Index	76.3	77.1	76.4	74.5	2.24	0.523	0.556	0.982	<0.001	0.802
Sarcomere length, µm	1.8	1.8	1.8	1.8	0.02	0.107	0.975	0.609	-	-

^1^ CON: No added guanidinoacetic acid (GAA); GAA3: Addition of 0.3 g/kg of DM GAA; GAA6: Addition of 0.6 g/kg of DM GAA; GAA9: Addition of 0.9 g/kg of DM GAA. ^2^ SEM: Standard error of the mean. ^3^ L: linear effect; Q: quadratic effect; Deviation: Quadratic deviation; T × T: treatment × aging time interaction. ^4^ Warner-Bratzler shear force.

## Data Availability

All relevant data are presented within the paper.

## Supplementary Materials

The following supporting information can be downloaded at: https://www.mdpi.com/article/10.3390/vetsci12050425/s1, Table S1: Effect of time on carcass ultrasound measurements in Nellore bulls supplemented with or without GAA¹ during the growing phase; Table S2: Effect of time on carcass ultrasound measurements in Nellore bulls supplemented with or without GAA¹ during the finishing phase; Table S3: Effect of aging time on meat quality measurements in Nellore bulls supplemented with or without GAA^1^.

## Abbreviations

The following abbreviations are used in this manuscript:

ADF	Acid detergent fiber
ADG	Average daily gain
BFT	Carcass backfat thickness
BFTu	Ultrasound backfat thickness
BW	Body weight
CL	Cooking loss
CY	Carcass yield
DMI	Dry matter intake
EE	Ether extract
FE	Feed efficiency
FQ	Forequarter
FQY	Forequarter yield
GAA	Guanidinoacetic acid
HQ	Hindquarter
HQY	Hindquarter yield
L*	Lightness
NDF	Neutral detergent fiber
REA	Carcass ribeye area
REAu	Ultrasound ribeye area
a*	Redness value
RFTu	Ultrasound rump fat thickness
TDN	Total digestible nutrients
TF	Thin flank
TFY	Thin flank yield
WBSF	Warner–Bratzler shear force
b*	Yellowness value

## References

[1] Owens F.N., Gill D.R., Secrist D.S., Coleman S.W. (1995). Review of some aspects of growth and development of feedlot cattle. J. Anim. Sci..

[2] Detmann E. (2022). Produziu o pasto para a seca. Como aproveitá-lo melhor usando a suplementação?. Todo ano tem seca! Está preparado? Estratégias para produção e uso do pasto na época seca.

[3] De Paula N.F., Paulino M.F., Couto V.R.M., Detmann E., Maciel I.F.S., Barros L.V., Lopes S.A., Valente É.E.L., Zervoudakis J.T., Martins L.S. (2019). Effects of supplementation plan on intake, digestibility, eating behavior, growth performance, and carcass characteristics of grazing beef cattle. Semin.-Cienc. Agrar..

[4] MacLaren D. (2005). Creatine. Drugs in Sport.

[5] Nelson D.L., Cox M.M. (2014). Princípios de Bioquímica de Lehninger.

[6] Owens F.N., Dubeski P., Hanson C.F. (1993). Factors that alter the growth and development of ruminants. J. Anim. Sci..

[7] Oliveira I.M.d., Paulino P.V.R., Marcondes M.I., Valadares Filho S.d.C., Detmann E., Cavali J., Duarte M.d.S., Mezzomo R. (2011). Pattern of tissue deposition, gain and body composition of Nellore, F1 Simmental× Nellore and F1 Angus× Nellore steers fed at maintenance or ad libitum with two levels of concentrate in the diet. Rev. Bras. Zootec..

[8] Hargreaves M., Spriet L.L. (2020). Skeletal muscle energy metabolism during exercise. Nat. Metab..

[9] Baker S.A., Gajera C.R., Wawro A.M., Corces M.R., Montine T.J. (2021). GATM and GAMT synthesize creatine locally throughout the mammalian body and within oligodendrocytes of the brain. Brain Res..

[10] Edison E.E., Brosnan M.E., Meyer C., Brosnan J.T. (2007). Creatine synthesis: Production of guanidinoacetate by the rat and human kidney in vivo. Am. J. Physiol-Ren..

[11] Ostojic S.M. (2021). Creatine synthesis in the skeletal muscle: The times they are a-changin. Am. J. Physiol. Endocrinol. Metab..

[12] Ostojic S.M. (2021). Safety of dietary guanidinoacetic acid: A villain of a good guy?. Nutrients.

[13] Guimarães-Ferreira L. (2014). Role of the phosphocreatine system on energetic homeostasis in skeletal and cardiac muscles. Einstein.

[14] Farshidfar F., Pinder M.A., Myrie S.B. (2017). Creatine supplementation and skeletal muscle metabolism for building muscle mass-review of the potential mechanisms of action. Curr. Protein Pept. Sci..

[15] Leite M.O., Knifis F.W., Machado M. (2023). Creatine supplementation and Akt/mTOR pathway: Unraveling the connection for optimal muscle performance. J. Sports Med. Ther..

[16] Ahmadipour B., Khajali F., Sharifi M.R. (2018). Effect of guanidinoacetic acid supplementation on growth performance and gut morphology in broiler chickens. Poult. Sci. J..

[17] Lu Y., Zou T., Wang Z., Yang J., Li L., Guo X., He Q., Chen L., You J. (2020). Dietary guanidinoacetic acid improves the growth performance and skeletal muscle development of finishing pigs through changing myogenic gene expression and myofibre characteristics. J. Anim. Physiol. Anim. Nutr..

[18] Valini G.A.d.C., Duarte M.d.S., Rodrigues G.d.A., Veroneze R., Saraiva A., Hausman G., Rocha G.C. (2020). Guanidinoacetic acid supplementation on growth performance and molecular mechanisms of lean mass gain in nursery pigs. Cienc. Rural.

[19] Zhu Z., Gu C., Hu S., Li B., Zeng X., Yin J. (2020). Dietary guanidinoacetic acid supplementation improved carcass characteristics, meat quality and muscle fibre traits in growing-finishing gilts. J. Anim. Physiol. Anim. Nutr..

[20] Li S.Y., Wang C., Wu Z.Z., Liu Q., Guo G., Huo W.J., Zhang J., Chen L., Zhang Y.L., Pei C.X. (2020). Effects of guanidinoacetic acid supplementation on growth performance, nutrient digestion, rumen fermentation and blood metabolites in Angus bulls. Animal.

[21] Liu C., Wang C., Zhang J., Liu Q., Guo G., Huo W.J., Pei C.X., Chen L., Zhang Y.L. (2021). Guanidinoacetic acid and betaine supplementation have positive effects on growth performance, nutrient digestion and rumen fermentation in Angus bulls. Anim. Feed Sci. Tech..

[22] Liu Y.J., Chen J.Z., Wang D.H., Wu M.J., Zheng C., Wu Z.Z., Wang C., Liu Q., Zhang J., Guo G. (2021). Effects of guanidinoacetic acid and coated folic acid supplementation on growth performance, nutrient digestion and hepatic gene expression in Angus bulls. Br. J. Nutr..

[23] Barioni L.G., Ferreira A.C. (2007). Monitoramento da Massa de Forragem e Altura Para Ajustes de Taxa de Lotação em Fazenda Agropecuária na Região do Cerrado. Embrapa Cerrados.

[24] AOAC (1990). Official Methods of Analysis.

[25] Weiss W.P., Conrad H.R., St. Pierre N.R. (1992). A theoretically-based model for predicting total digestible nutrient values of forages and concentrates. Anim. Feed Sci. Tech..

[26] National Academies of Sciences, Engineering, and Medicine (2016). Nutrient Requirements of Beef Cattle: Eighth Revised Edition.

[27] AOAC International (2005). Official Methods of Analysis.

[28] Van Soest P.J., Robertson J.B., Lewis B.A. (1991). Methods for dietary fiber, neutral detergent fiber, and nonstarch polysaccharides in relation to animal nutrition. J. Dairy Sci..

[29] Van Soest P.J. (1963). Use of Detergents in the Analysis of Fibrous Feeds. II. A Rapid method for the determination of fiber and lignin. J. Assoc. Off. Agric. Chem..

[30] Archer J.A., Bergh L. (2000). Duration of performance tests for growth rate, feed intake and feed efficiency in four biological types of beef cattle. Livest. Prod. Sci..

[31] Silva S.d.L.e., Tarouco J.U., Ferraz J.B.S., Gomes R.d.C., Leme P.R., Navajas E.A. (2012). Prediction of retail beef yield, trim fat and proportion of high-valued cuts in Nellore cattle using ultrasound live measurements. Rev. Bras. Zootec..

[32] Brasil (1997). Regulamento da Inspeção Industrial e Sanitária de Produtos de Origem Animal [Food Ofanimal Origin Sanitary and Industry Inspection]. https://www.gov.br/agricultura/pt-br/assuntos/inspecao/produtos-animal/arquivos/TITULOS_E_SUBTITULOS_SETEMBRO_09_13.pdf.

[33] Hunt M.C. (1980). Guidelines for Meat Color Evaluation.

[34] AMSA (1995). Research Guidelines for Cookery, Sensory Evaluation, and Instrumental Tenderness Measurements of Fresh Meat.

[35] AOAC (2007). AOAC Official Method 2007.04: Fat, Moisture, and Protein in Meat and Meat Products. Official Methods of Analysis.

[36] Culler R., Smith G., Cross H. (1978). Relationship of myofibril fragmentation index to certain chemical, physical and sensory characteristics of bovine longissimus muscle. J. Food Sci..

[37] Ramos P.M., Santos-Donado P.R.d., Oliveira G.M.d., Contreras–Castillo C.J., Scheffler T.L., Silva S.d.L.e., Martello L.S., Delgado E.F. (2022). Beef of Nellore cattle has limited tenderization despite pH decline in Longissimus lumborum. Sci. Agric..

[38] Cross H., West R., Dutson T. (1981). Comparison of methods for measuring sarcomere length in beef semitendinosus muscle. Meat Sci..

[39] Koolmees P.A., Korteknie F., Smulders F.J. (1986). Accuracy and utility of sarcomere length assessment by laser diffraction. Food Struct..

[40] Morales Gómez J.F., Antonelo D.S., Beline M., Pavan B., Bambil D.B., Fantinato-Neto P., Saran-Netto A., Leme P.R., Goulart R.S., Gerrard D.E. (2022). Feeding strategies impact animal growth and beef color and tenderness. Meat Sci..

[41] Carvalho V.V., Paulino M.F., Detmann E., Chizzotti M.L., Martins L.S., Silva A.G., Lopes S.A., Moura F.H. (2017). Effects of supplements containing different additives on nutritional and productive performance of beef cattle grazing tropical grass. Trop. Anim. Health Prod..

[42] Geng Q., Lin W., Yang L., Hu X., Qiu X. (2025). Rumen-protected guanidinoacetic acid improves growth performance in beef cattle under chronic heat stress by reshaping gut microbiota and modulating serum metabolism. Front. Microbiol..

[43] Oviedo-Rondón E.O., Córdova-Noboa H.A. (2020). The Potential of guanidino acetic acid to reduce the occurrence and severity of broiler muscle myopathies. Front. Physiol..

[44] Wyss M., Kaddurah-Daouk R. (2000). Creatine and creatinine metabolism. Physiol. Rev..

[45] Shi K., Zhao H., Xu S., Han H., Li W. (2021). Treatment efficacy of high-dose creatine supplementation in a child with creatine transporter (SLC6A8) deficiency. Mol. Genet. Genom. Med..

[46] Snow R.J., Murphy R.M. (2001). Creatine and the creatine transporter: A review. Mol. Cell Biochem..

[47] Yi S., Hu S., Wang J., Abudukelimu A., Wang Y., Li X., Wu H., Meng Q., Zhou Z. (2024). Effect of guanidinoacetic acid supplementation on growth performance, rumen fermentation, blood indices, nutrient digestion, and nitrogen metabolism in Angus steers. Animals.

[48] Ministério da Agricultura, Pecuária e Abastecimento (2009). Instrução Normativa n° 41, de 17 de Dezembro de 2009. https://www1.tce.pr.gov.br/conteudo/instrucao-normativa-n-40-de-17-de-dezembro-de-2009/237433/area/249.

[49] Speer H.F., Pearl K.A., Titgemeyer E.C. (2020). Relative bioavailability of guanidinoacetic acid delivered ruminally or abomasally to cattle. J. Anim. Sci..

[50] Ostojic S.M. (2016). Guanidinoacetic acid as a performance-enhancing agent. Amino Acids.

[51] Russell B.J. (2002). Rumen Microbiology and Its Role in Ruminant Nutrition.

[52] Van Soest P.J. (1994). Nutritional Ecology of the Ruminant.

[53] Ardalan M., Miesner M.D., Reinhardt C.D., Thomson D.U., Armendariz C.K., Smith J.S., Titgemeyer E.C. (2021). Effects of guanidinoacetic acid supplementation on nitrogen retention and methionine flux in cattle. J. Anim. Sci..

[54] Riesberg L.A., Weed S.A., McDonald T.L., Eckerson J.M., Drescher K.M. (2016). Beyond muscles: The untapped potential of creatine. Int. Immunopharmacol..

[55] Brosnan J.T., Brosnan M.E. (2010). Creatine metabolism and the urea cycle. Mol. Genet. Metab..

[56] Ostojic S.M., Niess B., Stojanovic M., Obrenovic M. (2013). Co-administration of methyl donors along with guanidinoacetic acid reduces the incidence of hyperhomocysteinaemia compared with guanidinoacetic acid administration alone. Br. J. Nutr..

[57] Ostojic S.M., Stojanovic M., Drid P., Hoffman J.R. (2014). Dose–response effects of oral guanidinoacetic acid on serum creatine, homocysteine and B vitamins levels. Eur. J. Nutr..

[58] Alonso L., Maquivar M., Galina C.S., Mendoza G.D., Guzmán A., Estrada S., Villareal M., Molina R. (2008). Effect of ruminally protected Methionine on the productive and reproductive performance of grazing Bos indicus heifers raised in the humid tropics of Costa Rica. Trop. Anim. Health Prod..

[59] Richardson C.R., Hatfield E.E. (1978). The limiting amino acids in growing cattle. J. Anim. Sci..

[60] Crouse M.S., Cushman R.A., Redifer C.A., Neville B.W., Dahlen C.R., Caton J.S., Diniz W.J.S., Ward A.K. (2024). International Symposium on Ruminant Physiology: One-carbon metabolism in beef cattle throughout the production cycle. J. Dairy Sci..

[61] Li Z., Liang H., Xin J., Xu L., Li M., Yu H., Zhang W., Ge Y., Li Y., Qu M. (2021). Effects of dietary guanidinoacetic acid on the feed efficiency, blood measures, and meat quality of Jinjiang bulls. Front. Vet. Sci..

[62] Eckhardt E.P., Kim W., Jaborek J., Garmyn A.J., Kang D., Kim J. (2024). Evaluation of guanidinoacetic acid supplementation on finishing beef steer growth performance, skeletal muscle cellular response, and carcass characteristics. J. Anim. Sci..

[63] Carvalho J.R.R., Schoonmaker J.P., Chizzotti M.L., Teixeira P.D., Dias J.C.O., Gionbelli T.R.S., Rodrigues A.C., Costa S.F., Ladeira M.M. (2019). Total nutrient digestibility and small intestine starch digestion in Nellore and Angus young bulls fed a whole shelled corn diet. J. Anim. Physiol. Anim. Nutr..

[64] Yi S., Ye B., Wang J., Yi X., Wang Y., Abudukelimu A., Wu H., Meng Q., Zhou Z. (2024). Investigation of guanidino acetic acid and rumen-protected methionine induced improvements in longissimus lumborum muscle quality in beef cattle. Meat. Sci..

[65] Li X., Liu X., Song P., Zhao J., Zhang J., Zhao J. (2022). Skeletal muscle mass, meat quality and antioxidant status in growing lambs supplemented with guanidinoacetic acid. Meat Sci..

[66] Li W.-J., Jiang Y.-W., Cui Z.-Y., Wu Q.-C., Zhang F., Chen H.-W., Wang Y.-L., Wang W.-K., Lv L.-K., Xiong F.-L. (2023). Dietary guanidine acetic acid addition improved carcass quality with less back-fat thickness and remarkably increased meat protein deposition in rapid-growing lambs fed different forage types. Foods.

[67] Francaux M., Poortmans J.R. (1999). Effects of training and creatine supplement on muscle strength and body mass. Eur. J. Appl. Physiol. O.

[68] Häussinger D., Roth E., Lang F., Gerok W. (1993). Cellular hydration state: An important determinant of protein catabolism in health and disease. Lancet.

[69] Bowker B., Petracci M., Berri C. (2017). Developments in Our Understanding of Water-Holding Capacity. Poultry Quality Evaluation.

[70] Fennema O.R. (1996). Food Chemistry.

