# Supplementary material for: Guanidinoacetic Acid and Its Impact on the Performance, Carcass and Meat Quality of Growing and Finishing Nellore Cattle
Source: Vet Sci. 2025 Apr 30;12(5):425. doi: 10.3390/vetsci12050425 (PMC12115749; doi:10.3390/vetsci12050425)
Supplement: Supplementary file 1 [file vetsci-12-00425-s001.zip › vetsci-3592982-supplementary.pdf]

Supplementary Material

Table S1. Effect of time on carcass ultrasound measurements in Nellore bulls supplemented with or without GAA[1] during the growing phase.

Item[3]	Time[2]					SEM	P-value
	1	70	139	203	280		
REAu, cm^2	42.2	47.4	50.6	61.2	68.3	0.68	<0.001
BFTu, mm	0.40	0.44	0.86	1.68	2.92	0.15	<0.001
RFTu, mm	0.93	2.05	2.43	3.27	4.71	0.21	<0.001

[1] GAA = Guanidinoacetic acid. [2] The reported means represent the average of the four treatments at each time point. [3] REAu: ultrasound ribeye area; BFTu: ultrasound backfat thickness; RFTu: ultrasound rump fat thickness.

Table S2. Effect of time on carcass ultrasound measurements in Nellore bulls supplemented with or without GAA[1] during the finishing phase.

Item[3]	Time[2]			SEM	P-value
	1	58	78		
REAu, cm^2	68.6	80.0	89.9	0.74	<0.001
BFTu, mm	2.88	4.26	5.07	0.19	<0.001
RFTu, mm	4.67	6.13	7.29	0.29	<0.001

[1] GAA = Guanidinoacetic acid. [2] The reported means represent the average of the four treatments at each time point. [3] REAu: ultrasound ribeye area; BFTu: ultrasound backfat thickness; RFTu: ultrasound rump fat thickness.

Table S3. Effect of aging time on meat quality measurements in Nellore bulls supplemented with or without GAA[1].

Item	Aging time[2]				SEM	P-value
	No aging	7	14	21		
Color						
Redness (a*)	21.29	23.88	23.18	23.09	0.36	<0.001
Yellowness (b*)	12.54	16.98	16.33	15.87	0.41	<0.001
Cooking loss, %	28.36	27.90	26.23	27.32	0.28	<0.001
WBSF[3], N	74.00	60.48	51.06	45.99	14.8	<0.001
Myofibrillar Fragmentation Index	56.38	64.27	88.89	95.53	2.04	<0.001

[1] GAA = Guanidinoacetic acid. [2] The reported means represent the average of the four treatments at each aging time point. [3] Warner-Bratzler shear force
